# Supplementary material for: A set of multi-entry identification keys to African frugivorous flies (Diptera, Tephritidae)
Source: Zookeys. 2014 Jul 24;(428):97–108. doi: 10.3897/zookeys.428.7366 (PMC4143993; doi:10.3897/zookeys.428.7366)
Supplement: Supplementary material 6 — Key to Ceratitis [file zookeys-428-097-s006.zip › SF6_ZooKeys_key to Ceratitis/key/SF6_ZooKeys_key to Ceratitis/Media/Html/Ceratitis obtusicuspis.htm]

Ceratitis (Pterandrus) obtusicuspis, n


***Ceratitis*** **(*Pterandrus*) *obtusicuspis* De Meyer & Freidberg**

Body
length: 5.43 (5.00-5.80) mm; wing length: 5.95 (5.60-6.30) mm.

Male.

Head: Antenna yellow. First flagellomere three times as long
as pedicel. Arista with short to moderately long rays; only basally rays
slightly shorter and sparser ventrally. Frons pale yellow, in center slightly
darker; with short scattered setulae distinctly darker than frons. Frontal
setae well developed, sometimes posterior frontal partly reduced. Face whitish,
gena darker yellow. Genal seta and setulae black, well developed.

Thorax: Postpronotal
lobe white, without spot. Scutal pattern: ground color grayish-brown, with
orange tinge; with streaks and darker markings but without distinct spots or
clearly defined stripes, except shiny brown to brownish black sutural and
dorsocentral spots; prescutellar white markings separate. Scapular setae black.
Scutellum yellowish white, basally without spots, apically with three separate
black spots, extending to basal 0.33, sometimes along apical margins
intermediate area darker yellowish brown. Anepisternum with only ventral margin
darker yellow; setulae pale.

Legs:
Yellow except where otherwise noted; setation typical for subgenus, mainly
dark. Foreleg: femur somewhat darkened; posteriorly with dispersed long dark
setulae along entire length, but not forming distinct bush; ventral setae dark.
Midleg: femur largely orange-brown except distally; ventrally with dark short
feathering along distal 0.33, basally with more dispersed dark setulae; tibia
broadened, silvery shine when viewed from certain angle; with black feathering
dorsally along distal 0.8 to 0.9 and ventrally along distal 0.66, anteriorly
with dispersed dark setulae, one row longer setulae. Hindleg: femur at apical
0.25 with longer setulae dorsally and ventrally.

Wing:
banding yellowish brown. Interruption between marginal and discal bands
near vein R_1_ clear and complete; cubital band free; medial band
usually absent, sometimes weakly present; crossvein R-M opposite middle of discal
cell. Apex of vein R_1_ distal to level of crossvein R-M. Crossvein
DM-Cu oblique anterobasally.

Abdomen:
Mostly yellow. Tergite 1 with small black patches across posterior margin.
Tergites 2 and 4 with pale gray band on posterior 0.66; tergite 4 with anterior
0.33 brown. Tergite 3 completely or largely brown; tergite 5 with brown band
along anterior half, posterior margin narrowly brown. Brown bands on tergites
3, 4 and 5 often partly interrupted in middle. Male epandrium in lateral view
with posterior lobe of lateral surstylus stout and short, anterior lobe
pronounced.

Female

As
male except for the following characters: First flagellomere more yellowish
orange. Frons darker yellow. Anepisternum sometimes with few dark setulae along
ventral margin. Legs without feathering, yellow with femora slightly darker;
forefemur posteroventrally with dark pilosity. Abdominal tergite 5 across
posterior margin yellow. Oviscape shorter than preabdomen. Aculeus at most six
times longer than wide; tip blunt and broadened, with slight apical indentation
and lateral margin slightly sinuous.

(Description
after De Meyer & Freidberg, 2006)
